# Self-regulated learning and critical reflection in an e-learning on patient safety for third-year medical students

**DOI:** 10.5116/ijme.5b39.d5a8

**Published:** 2018-07-12

**Authors:** Rainer Gaupp, Götz Fabry, Mirjam Körner

**Affiliations:** 1 Freiburg University, Medical Faculty, Medical Psychology and Medical Sociology, Germany

**Keywords:** Critical reflection, self-regulated learning, e-learning, patient safety, evaluation

## Abstract

**Objectives:**

To explore the influence of critical thinking, self-regulated learning and system usability on the acceptance of e-learning on patient safety.

**Methods:**

A cross-sectional study was conducted, using a 32-question online survey. One hundred ninety-three (n=193 medical students participated in the study and were asked to rate levels of reflective thinking, self-regulated learning and attitudes towards patient safety using scales from the Questionnaire for Reflective Thinking, the Attitudes to Patient Safety Questionnaire and the System Usability Scale. Differences between reflection levels were calculated using paired t-tests, associations between critical thinking and self-regulated learning were calculated using linear correlations. We performed linear multiple regression analysis to identify predictors for student acceptance of the e-learning.

**Results:**

Students (n=193) engaged in intermediate levels of reflection (5-point Likert, M=3.62, SD=0.73) and significantly (t_(143)_=15.15, p<0.001, d=1.57) lower levels (M=2.35, SD=0.87) of critical reflection. Most students showed high (≥ 4; 44.1%) or intermediate (<4 level > 2; 29.4 %) levels of self-regulated learning. A regression model indicated that 5 predictors (Reflection, critical reflection, self-regulated learning, relevance, usability) explained 65.3% of the variance (R²=0.653, F_(5, 96)_=39.02, p<0.01) of perceived total quality.

**Conclusions:**

This study shows that reflection and learning skills are important factors for e-learning acceptance, but perceived relevance and system usability play a more important role. From a didactic perspective, it is indispensable to provide the students with sufficient examples and links to professional practice to enhance the perception of relevance and to improve system usability permanently.

## Introduction

E-learning has become a standard teaching approach in medical education.^1^ As part of the educational process, e-learning possesses characteristics that have the potential to improve upon traditional teaching methods: E-learning courses typically offer increased flexibility, adaptability, and student centeredness compared with traditional lectures, and provide far-reaching opportunities for collaboration and discussion.^2^ Thus, e-learning programs offer students the opportunity to engage in self-regulated and interactive learning at their own pace.

Increasingly, e-learning is being used to teach and improve patient safety,^3^ which is a primary goal of healthcare systems worldwide.^4^ To enhance patient safety, both researchers and practitioners recommend (among a diversified set of actions) a change in the organizational culture^5^ towards a culture of safety. Such cultural changes require individuals’ willingness and ability to deeply reflect on actions, values, and beliefs^6^ to develop new mental models in which safety is central.^7^ An important foundation for developing a culture of safety involves acquiring specific knowledge about elements of patient safety. Given the significance of this knowledge, and of individual reflection, values, and beliefs, e-learning programs that provide ample opportunities for self-regulated learning may indeed be especially suited to prepare students for subsequent practice-based learning experiences related to patient safety (e.g., simulations). The abilities to reflect on a situation, the self and the learning process are not only essential to self-regulated learning,^8^ but are also fundamental for successful experiential learning^9^ through simulation exercises^10^ or bedside learning.

### Self-regulated learning and the perception of relevance

Self-regulated learning strategies can be considered key assets in guiding the individual learning process,^11^ as they show positive correlations with academic success in e-learning settings.^12^ For self-regulated learning, the learner must regulate a set of three layers: 1) the choice of cognitive strategies; 2) the use of metacognitive knowledge; and, 3) the choice of goals and resources deployed for learning.^13^ The first layer (choice of cognitive strategies) is largely influenced by motivational factors and the perception of the relevance of the topic.^14^ In this regard, technical aspects of system usability^15^ and content-related aspects of the course will have a great influence on this layer of self-regulated learning, as they both influence the learners’ motivation to complete an online course.^16^  The use of metacognitive knowledge requires reflection on one’s own learning process,^17^ and thus critical thinking.^18^ Critical thinking helps self-regulated learners to structure their learning process (third layer: regulation of the self) and thus facilitate deep processing of new knowledge.

### E-Learning Patient Safety (ELPAS)

Against the background of self-directed learning, we developed an interactive e-learning course for third-year medical students to acquire basic competencies in patient safety. Based on the learning objectives that were developed by the World Health Organization4 we created a course titled E-Learning Patient Safety (ELPAS), which focused on two major aspects: teamwork and error management. The course consisted of two modules. Module 1 focused on teamwork, and included both scientific background (e.g., Big Five in Teamwork)^19^ and practical application via video case studies. Module 2 addressed the field of error management, grounded in the work of Reason.^6^^,^^20^^,^^21^ In the second module, students had to apply theoretical knowledge in case studies using the London Protocol.^22^ Our course used differentiated content (e.g., journal articles, videos, interactive quizzes, and podcasts) to provide patient safety knowledge. Unlike a traditional lecture (or recorded lecture), learning with ELPAS focused on self-regulation: Users could decide individually on learning time, learning speed, and which content they used to study. Students worked in small groups and used Web 2.0 technology (e.g., etherpads and discussion boards) for discussions with peers and tutors. Online quizzes enriched the learning material to allow frequent self-assessment. Additionally, students were required to complete a set of five assignments. Two of the assignments were classical multiple-choice tests, while the other three were specifically designed to foster reflection. In two of these three assignments, students collaborated in groups of six and developed solutions for case studies in which they had to reflect on real cases presented to them as a report or short video. The third of these three assignments required critical peer feedback on their recensions of a scientific paper on patient safety. Thus, to achieve the learning goals, students had to engage in self-regulated learning to master the basic knowledge, which they had then to apply interactively to a number of case studies. Both online modules were available on the learning management system of the university, which is used throughout the school, and thus well known to the students. ELPAS was mandatory for all third-year medical students at our university.

### Aim and research hypotheses

The study aimed to identify educational and technical variables that help to explain students’ acceptance of the ELPAS course. Based on self-regulated learning theory, we hypothesized that: a) students who showed higher levels of critical thinking (reflection and critical reflection) would engage more in self-regulated learning, and b) students who showed more self-regulated learning behaviors would feel more comfortable with the course and thus rate ELPAS better than those who engaged less in self-regulated learning. Furthermore, we expected that: c) there would be an association between the perceived relevance of the topic and evaluation results. Following these hypotheses, we explored which predictors were relevant for e-learning acceptance by medical students. Referring to Davids and colleagues,^23^ we also expected that: d) evaluation results would be influenced by the perceived usability of the e-learning system. Clarification of these hypotheses may help to guide future developments in the field of e-learning.

## Methods

### Study design and participants

This was a cross-sectional survey study, using a 32-question online survey. A total of 193 medical students (57% of invited participants) participated in the study, 68% of whom were female. Most participants were between 20 and 29 years old (89%), and only some of them (21%) had prior experience in a healthcare profession such as nursing or pre-hospital care. Age and sex distributions of the sample were comparable to the total population in this cohort (338 students). As participation was voluntary, and no incentive was offered for participation, the response rate was within the expected range.^24^

The study was planned and conducted in compliance with the Declaration of Helsinki on ethical principles for research involving human subjects. Participants were informed about the goals of the study and were told that participation was completely voluntary. Participants were informed that they could terminate their participation at any time without any negative consequences. The survey was completely anonymous (i.e., no personally identifiable data was collected, retrieved, or reviewed at any stage of our analysis). The Ethics Committee of Freiburg University approved the protocol.

### Data collection method

The survey was administered to 338 third-year medical students through personalized links. We used a third-party survey tool to guarantee anonymity. The survey contained scales for self-regulated learning, reflective thinking, perceived relevance, and system usability. The self-regulated learning scale contained four items (e.g., “Through ELPAS, I could monitor my learning progress easily”) on a 5-point Likert scale ranging from 1 (“do not agree”) to 5 (“fully agree”) and was adapted from Reinhard,^25^ there were no prior psychometrics available for this scale. The reflective thinking scale measured both reflection (four items; e.g., “I sometimes question the way others do something and try to think of a better way”) and critical reflection (four items; e.g., “This course has challenged some of my firmly held ideas”) on a 5-point Likert scale ranging from 1 (“do not agree”) to 5 (“fully agree”). The scale was developed and tested by Kember and colleagues,^18^ and showed acceptable factor structure in a confirmatory factor analysis (c²=179.3, df=100, CFI = 0.903) and moderately strong reliability (reflection: ɑ=0.63, critical reflection: ɑ=0.68). To measure perceived relevance (two items: “Teaching students about patient safety should be an important priority in medical students training”; “Learning about patient safety issues before I qualify will enable me to become a more effective doctor”), a subscale from the German Attitudes to Patient Safety Questionnaire (GAPSQ)^26^ was used; its reliability was tested by the original authors (ɑ = 0.74). To measure system usability, we used the robust System Usability Scale (SUS).^27^^,^^28^ Students also rated the quality of nine elements of the e-learning on a 5-point Likert scale ranging from 1 (“I think it is very bad”) to 5 (“I think it is very good”). As we were interested in the perception of the overall quality of the ELPAS course, we summed the single ratings and calculated their mean as an overall rating of the course. Prior to calculating the means for self-regulated learning, reflective thinking, perceived relevance, system usability, and total quality of the e-learning course, we analyzed the reliability of the scales using Cronbach’s alpha. All relevant scales (see Table 1) showed acceptable to good reliability.^29^

### Statistical analysis

SPSS version 24 was used for statistical analysis. Prior to statistical analysis, the items were checked for plausibility, and missing data analysis was performed using the expectation-maximization algorithm (p>0.05). No item was eliminated from the analysis because of missing data. Differences between participant-reported reflection and critical reflection were tested for significance using paired t-tests.  Associations between critical thinking and self-regulated learning were calculated using linear correlations. We expected that perceived relevance, critical thinking, and self-regulated learning would explain large amounts of the total variance on overall rating and accordingly established a linear multiple regression model. Suspecting that system usability might be an important additional predictor,^23^ we included the usability results in the model. Significance for all tests was set at p<0.05. Effect sizes were computed according to Cohen’s d.

## Results

In total, students rated the online course as “average” (M= 2.93, SD = 0.8) on the 5-point Likert scale for overall rating (1 = “I think it’s very bad”; 5 = “I think it’s very good”) and perceived the objectives as “relevant” (M = 5.41, SD = 1.42) on the 7-point Likert scale (1 = “don’t agree at all”, 7 = “fully agree”). While we did not evaluate the pre-existing computer skills of the participants, we did check whether the participants were overtaxed by the technical requirements or the content-wise requirements of the e-learning by using a 5-point Likert scale (1= “requirements are too low for me”; 3 = “fits very well”, 5 =“requirements are too high for me”). Students reported that the course fit well with their abilities with respect to content (M=3.13, SD= 0.63) and technical requirements (M=3.30, SD=0.64).

The 5-point Likert scales measured the overall levels of reflective thinking for reflection and critical reflection (5 = highest level). While most of the students showed intermediate to high levels of reflection when doing the online course (M=3.65, SD=0.73), the levels of critical reflection were significantly (t_(__143)_ = 15.15, p<0.001, d=1.57) lower (M= 2.38, SD = 0.88). Most students reported high (≥ 4; 44.1%) or intermediate (< 4 level > 2; 29.4 %) levels of self-regulated learning, while 26.6 % showed low self-regulated learning levels (≤ 2.0 on the 5-point scale). The results show a moderate correlation between reflective thinking and self-regulated learning (r=0.49, p<0.001), and a strong correlation between self-regulated learning and overall rating (r=0.61, p<0.001). See Table 1 for an overview of scale items and measures.

Reflection, critical reflection, self-regulated learning, relevance perception, and system usability were entered in a multiple regression analysis to predict overall rating. The results of the regression indicated that the five predictors explained 65.3% of the variance (R²=0.653, F_(__5,96)_=39.02, p<0.01). Tests, if data met the assumption of collinearity, indicated that multicollinearity was not a relevant concern (relevance, reflection, critical reflection, self-regulated learning, system usability tolerance= 0.58–0.85, VIF = 1.5–1.73). Overall rating was primarily predicted by system usability (ß=.35, t_(__102)_=4.56, p<0.001), followed by perceived relevance (ß=.25, t_(102)_=3.27,p=0.001) and critical reflection (ß=.22, t_(102)_=2.94, p=0.004). To a lesser extent, self-regulated learning (ß=.17, t_(__102)_=2.17, p=0.032) also predicted overall rating. Reflection as the remaining predictor showed only small, statistically non-significant beta coefficients (ß=0.10, t_(__102)_=1.60, p=0.113). The raw and standardized regression coefficients of the predictors are shown in Table 2.

Hypotheses a, b, c, and d are supported by our study: students with high levels of reflection or critical reflection reported higher self-regulated learning behavior, and students with high self-regulation rated the course better than those with low self-regulation. 

**Table 1:** Descriptive results on the item-level and Cronbach’s Alpha for the scales

Scale	Item	Mean	SD	α
Self-regulated learning: 5-point Likert^25^		**2.93**	**1.04**	**0.81**
	ELPAS enabled me to arrange my learning time flexible.	3.76	1.23	
	Due to the introduction of ELPAS, I dealt with the content more intensively than in a traditional lecture.	2.66	1.38	
	Due to the introduction of ELPAS, I learned more independently than in a traditional lecture.	2.77	1.39	
	Through ELPAS, I could monitor my learning progress easily.	2.51	1.21	
Reflection: 5-point Likert^18^		**3.62**	**0.73**	**0.71**
	I sometimes question the way others do something and try to think of a better way.	2.68	1.21	
	I like to think over what I have been doing and consider alternative ways of doing it.	3.96	0.92	
	I often reflect on my actions to see whether I could have improved on what I did.	3.87	0.93	
	I often reappraise my experience, so I can learn from it and improve for my next performance.	3.95	0.89	
Critical reflection: 5-point Likert^18^		**2.35**	**0.87**	**0.77**
	As a result of this course, I have changed the way I look at myself.	2.34	1.17	
	ELPAS has challenged some of my firmly held ideas.	2.23	1.14	
	As a result of ELPAS, I have changed my normal way of doing things.	2.72	1.17	
	During this course, I discovered faults in what I had previously believed to be right.	2.23	1.03	
Relevance: 7-point Likert^26^		**5.41**	**1.42**	**0.90**
	Teaching students about patient safety should be an important priority in medical students training.	5.47	1.42	
	Learning about patient safety issues before I qualify will enable me to become a more effective doctor.	5.36	1.54	

Note: Figures in bold represent aggregated results of each scale

The perceived relevance of the topic of patient safety was an important predictor for overall rating. Additionally, as hypothesized, perceived system usability influenced ratings of the total quality of the e-learning course.

**Table 2:** Regression analysis

Model	B	SE_B_	ß	t	Sig.
Constant	0.279	0.259		1.077	0.284
Relevance	0.133	0.041	0.251	3.273	0.001
Reflection	0.113	0.070	0.103	1.603	0.112
Critical reflection	0.192	0.065	0.215	2.944	0.004
Self-regulated learning	0.126	0.058	0.174	2.174	0.032
System Usability	0.013	0.003	0.353	4.556	0.000

Note: Independent variable was overall rating “total quality of the E-Learning course”. R2=.670, adjusted R2=.653

## Discussion

With this study, we tried to understand better, how educational variables predict students’ overall rating of the ELPAS course. Our analysis shows that most students engage in reflective thinking while taking the ELPAS course, whereas critical reflection is employed to a much lower extent. Reflection itself can be considered a self-regulated learning activity,^8^ and is associated with self-regulated learning in our data, however the associations remain rather weak. This might be explained by individual motivation: Even if students can reflect and do reflect, they might not be motivated to engage deeply in self-regulated learning.^13^ Although we did not measure motivation, we did measure the level of perceived relevance, which correlates significantly with reflection, and, therefore, arguably relates to the level of self-regulated learning.

Concerning Mezirow’s^30^ transformative learning theory, students will adopt significant changes of perspectives^18^ and thus engage in deeper knowledge processing strategies only when their level of reflection deepens towards critical reflection. Our analysis showed a pronounced association between critical reflection and self-regulated learning, indicating a medium-to-large effect size.^29^ In Boekaerts’^13^ three-layered model of self-regulated learning, critical reflection is essential when regulating the self and defining individual goals (i.e., the outer layer of the model).With hypotheses b, c, and d, we aimed to understand predictors for total quality outcomes of the e-learning course, from an educational and instructional design perspective. The influence of self-regulated learning in the model was less pronounced than expected, but was still detectable and statistically significant, suggesting that self-regulation capabilities are important for succeeding in e-learning environments.^31^ The design of the e-learning system used in this study might help explain this: it facilitated self-regulated learning and metacognitive strategies by providing immediate feedback (monitoring strategies), linking relevant literature (resource strategies) and allowing discussions with peers (cooperation strategies). Hence, it supported those students who were unused to self-regulate their learning process independently. For students unable or unwilling to use cognitive and metacognitive strategies to improve their self-regulation, such online courses will have limited effects for either acquiring knowledge or develop beneficial attitudes towards patient safety.^32^

Whether students perceive the specific content of a didactic session as relevant for their individual learning goals depends on various factors.^33^ Particularly with novice medical students, prior experience in the field of practice,^34^ and thus better ability to rate the practical importance of content, might influence the perception of relevance. However, in our study, students’ perception of topical relevance did not differ significantly between students irrespective of prior experience in healthcare professions. While the perception of relevance did not differ between the two groups, our analysis showed that those students who considered the content relevant for their professional development rated the course better than those who found the content less relevant.^35^ Therefore, we argue from a didactic perspective that it is essential to provide students with sufficient examples and links to professional practice to enhance their perception of relevance.

Besides educational predictors, we expected system usability to be a major predictor^23^ for overall quality rating. Indeed, system usability was the most pronounced predictor for overall quality rating but also showed strong correlations with self-regulated learning. These results suggest that improving the usability of an e-learning course will not only satisfy more students but will also support learning.^36^^-^^38^

We evaluated a novel comprehensive e-learning course on patient safety. While several reports exist for evaluations of online courses that were conducted to teach specific skills around patient safety (e.g., safety in blood transfusions and dosage calculation),^3^^,^^39^^,^^40^ only very few exist in which the topic of patient safety was approached holistically by an online course.^41^ Typically, patient safety education uses more face-to-face teaching methods such as simulation, role-playing, discussions, or games.^42^ Our study showed that patient safety education using a distance-learning methodology was acceptable to a significant proportion of the population in question and that improving usability might increase students’ acceptance of such courses. Thus, e-learning courses may become a valuable part of patient safety education and may be considered as complementary to more traditional training methods such as simulation or role-playing.

### Limitations

This study measured the influences of self-regulated learning and various levels of reflection on students’ overall ratings of the quality of an e-learning course. Because the data collection was anonymized, objective indicators generated by the learning management system, including time logs or test results, were not available. Therefore, the outcome parameter was highly subjective and may have been influenced by several factors which are not accounted for in the study, and thus could not be controlled (e.g., individual learning preferences, prior experience with e-learning tools, and computer literacy). The generalizability of the results is limited because we only included medical students from Freiburg University, and the response rate of 57% was not optimal.

Moreover, the voluntary participation of the students may have introduced selection effects; it is also possible that motivated students were more likely to participate. The medium-to-strong correlations of the scales could be the result of overlapping constructs and common-method bias. We also relied on self-reported data, which may have given rise to single-source bias. Furthermore, the study’s design is cross-sectional and hence does not allow for a causal interpretation of the relationships found in the predictive model. Longitudinal or intervention studies should be conducted to examine the causality of the proposed relationships.

## Conclusions

Our results suggest that students engage in critical thinking when they study in our e-learning environment on patient safety. Thus, we believe that through a combination of different learning tasks, reflection and critical reflection can be fostered through e-learning. This could be used to prepare students for subsequent interactive face-to-face sessions. However, students will need a distinct set of self-regulated learning skills to maximize the benefit of such learning environments. While reflection and learning skills are important for successful student engagement with e-learning environments, perceived relevance and system usability also play an important role with respect to students’ acceptance of an online course. When designing novel e-learning environments, medical educators should set standards towards system usability, as this will significantly affect students learning experience with the e-learning course.

### Conflict of Interest

The authors declare that they have no conflict of interest.

## References

[1] George PP, Papachristou N, Belisario JM, Wang W, Wark PA, Cotic Z, Rasmussen K, Sluiter R, Riboli-Sasco E, Tudor Car L, Musulanov EM, Molina JA, Heng BH, Zhang Y, Wheeler EL, Al Shorbaji N, Majeed A, Car J (2014). Online eLearning for undergraduates in health professions: A systematic review of the impact on knowledge, skills, attitudes and satisfaction.. J Glob Health.

[2] Ellaway R, Masters K (2008). AMEE Guide 32: e-Learning in medical education Part 1: Learning, teaching and assessment.. Med Teach.

[3] McHugh SM, Corrigan M, Dimitrov B, Cowman S, Tierney S, Humphreys H, Hill A (2010). A targeted e-learning program for surgical trainees to enhance patient safety in preventing surgical infection.. J Contin Educ Health Prof.

[4] WHO. Patient safety curriculum guide. Geneva 2011[cited 26 February 2018]; Available from http://www.who.int/patientsafety/education/mp_curriculum_guide/en/.

[5] VanGeest J, Cummins D. An educational needs assessment for improving patient safety. National Patient Safety Foundation; 2003 [cited 20 February 2018]; Available from: https://cdn.ymaws.com/www.npsf.org/resource/collection/ABAB3CA8-4E0A-41C5-A480-6DE8B793536C/Educational_Needs_Assessment.pdf.

[6] Reason JT. Managing the risks of organizational accidents. Aldershot, Hants, England; Brookfield, Vt., USA: Ashgate; 1997.

[7] Vincent C. Patient safety. Chichester, West Sussex: Wiley-Blackwell; 2010.

[8] Sandars J (2009). The use of reflection in medical education: AMEE Guide No. 44.. Med Teach.

[9] Kolb DA. Experiential learning: Experience as the source of learning and development. Englewood Cliffs: Prentice-Hall; 1984.

[10] McGaghie WC, Issenberg SB, Petrusa ER, Scalese RJ (2010). A critical review of simulation-based medical education research: 2003-2009.. Med Educ.

[11] Sandars J, Cleary TJ (2011). Self-regulation theory: applications to medical education: AMEE Guide No. 58.. Med Teach.

[12] Broadbent J, Poon WL. Self-regulated learning strategies & academic achievement in online higher education learning environments: a systematic review. Internet and Higher Education. 2015;27:1-13.

[13] Boekaerts M. Self-regulated learning: where we are today. International Journal of Educational Research. 1999;31:445-547.

[14] Pintrich PR. The role of motivation in promoting and sustaining self-regulated learning. International Journal of Educational Research. 1999;31(6):459-70.

[15] Faghih b, Azadehfar, Katebi SD. User interface design for e-learning software. The International Journal of Soft Computing and Software Engineering. 2013;3(3):786+94.

[16] Junus IS, Santoso HB, Isal RYK, Utomo AY. Usability evaluation of the student centered e-Learning environment. International Review of Research in Open and Distributed Learning. 2015;16(4):62-82.

[17] Zimmerman BJ (2002). Becoming a Self-Regulated Learner: An Overview.. Theory Into Practice.

[18] Kember D, Leung DYP, Jones A, Loke AY, McKay J, Sinclair K, et al. Development of a questionnaire to measure the level of reflective thinking. Assessment & Evaluation in Higher Education. 2000;25(4):381-95.

[19] Salas E, Sims DE, Burke CS (2016). Is there a "Five" in Teamwork?. Small Group Research.

[20] Reason J. Human error. Cambridge: Cambridge University Press; 1990.

[21] Reason J. Safety paradoxes and safety culture. Inj Control Saf Promot. 2000;7(1):3-14.

[22] Taylor-Adams S, Vincent C. Systems analysis of clinical incidents. London: Imperial College London; 2005.

[23] Davids MR, Chikte UM, Halperin ML (2014). Effect of improving the usability of an e-learning resource: a randomized trial.. Adv Physiol Educ.

[24] Doring N, Bortz J. Forschungsmethoden und Evaluation in den Sozial- und Humanwissenschaften. 5. vollst. uberarb., aktualisierte und erw. Aufl. ed. Berlin: Springer; 2016.

[25] Grote B, Hoffmann H, Reinhardt J. e-Learning and Web 2.0 in the humanities - development, testing and evaluation of didactic models beyond the distribution of online-material. Proceedings of the 7th European Conference on e-Learning, 2008, Agia Napa, Cyprus. Reading: Academic Publishing International Ltd; 2008.

[26] Kiesewetter J, Kager M, Lux R, Zwissler B, Fischer MR, Dietz I (2014). German undergraduate medical students' attitudes and needs regarding medical errors and patient safety--a national survey in Germany.. Med Teach.

[27] Brooke J. SUS: A 'Quick and Dirty' Usability Scale. In: Jordan PW, Thomas B, McClelland L, Weerdmeester B, editors. Usability evaluation in industry. London: Taylor & Francis; 1996.

[28] Lewis JR, Sauro J. The factor structure of the system usability scale. In: Hutchison D, Kanade T, Kittler J, Kleinberg JM, Mattern F, Mitchell JC, et al., editors. Human Centered Design. Berlin, Heidelberg: Springer Berlin Heidelberg; 2009.

[29] Field AP. Discovering statistics using IBM SPSS statistics: and sex and drugs and rock 'n' roll. Los Angeles: Sage; 2013.

[30] Mezirow J. Fostering critical reflection in adulthood: a guide to transformative and emancipatory learning. San Francisco: Jossey-Bass Publishers; 1990.

[31] Kassab SE, Al-Shafei AI, Salem AH, Otoom S. Relationships between the quality of blended learning experience, self-regulated learning, and academic achieve-ment of medical students: a path analysis. Adv Med Educ Pract. 2015; 627-34. 10.2147/AMEP.S75830 PMC4293215 25610011

[32] Paechter M, Maier B, Macher D. Students' expectations of, and experiences in e-learning: Their relation to learning achievements and course satisfaction. Computers & Education. 2010;54(1):222-9.

[33] Frymier AB, Shulman GM (1995). "What's in it for me?": Increasing content relevance to enhance students' motivation.. Communication Education.

[34] Siebert H. Didaktisches Handeln in der Erwachsenenbildung: Didaktik aus konstruktivistischer Sicht. Augsburg: ZIEL; 2006.

[35] Hodges CB. Designing to motivate: motivational techniques to incorporate in E-Learning experiences. Journal of Interactive Online Learning. 2004;2(3):1-7.

[36] Meiselwitz G, Sadera WA. Investigating the connection between usability and learning outcomes in online learning environments. Journal of Online Learning and Teaching. 2008;4(2):234-42.

[37] Sandars J (2010). The importance of usability testing to allow e-learning to reach its potential for medical education.. Educ Prim Care.

[38] Zaharias P, Poylymenakou A (2009). Developing a usability evaluation method for e-Learning applications: beyond functional usability.. International Journal of Human-Computer Interaction.

[39] Graham JE (2015). Transfusion e-learning for junior doctors: the educational role of 'LearnBloodTransfusion'.. Transfus Med.

[40] Lee TY, Lin FY (2013). The effectiveness of an e-learning program on pediatric medication safety for undergraduate students: a pretest-post-test intervention study.. Nurse Educ Today.

[41] Evans AM, Ellis G, Norman S, Luke K (2014). Patient safety education - a description and evaluation of an international, interdisciplinary e-learning programme.. Nurse Educ Today.

[42] Gordon M, Darbyshire D, Baker P (2012). Non-technical skills training to enhance patient safety: a systematic review.. Med Educ.

